# LSTM algorithm optimization for COVID-19 prediction model

**DOI:** 10.1016/j.heliyon.2024.e26158

**Published:** 2024-02-16

**Authors:** Irwan Sembiring, Sri Ngudi Wahyuni, Eko Sediyono

**Affiliations:** a Satya Wacana Christian University, 50711, Salatiga, Indonesia; b Universitas Amikom Yogyakarta, 55581, Indonesia

**Keywords:** COVID-19, Time series prediction, LSTM model, Optimization

## Abstract

The development of predictive models for infectious diseases, specifically COVID-19, is an important step in early control efforts to reduce the mortality rate. However, traditional time series prediction models used to analyze the disease spread trends often encounter challenges related to accuracy, necessitating the need to develop prediction models with enhanced accuracy. Therefore, this research aimed to develop a prediction model based on the Long Short-Term Memory (LSTM) networks to better predict the number of confirmed COVID-19 cases. The proposed optimized LSTM (popLSTM) model was compared with Basic LSTM and improved MinMaxScaler developed earlier using COVID-19 dataset taken from previous research. The dataset was collected from four countries with a high daily increase in confirmed cases, including Hong Kong, South Korea, Italy, and Indonesia. The results showed significantly improved accuracy in the optimized model compared to the previous research methods. The contributions of popLSTM included 1) Incorporating the output results on the output gate to effectively filter more detailed information compared to the previous model, and 2) Reducing the error value by considering the hidden state on the output gate to improve accuracy. popLSTM in this experiment exhibited a significant 4% increase in accuracy.

## 1 Introduction

COVID-19 was reported as a global outbreak in 2019, causing significant health burdens in all countries worldwide. According to WHO, the number of confirmed cases as of March 2022 was 472,816,657, including 6,099,380 deaths globally [1]. The pandemic, attributed to the SARS-2 Virus [2], has significantly disrupted the pace of the world, causing a surge in infectious trends of unprecedented magnitude. It originated from Wuhan City, Hubei Province, China, and was first reported in December 2019. The outbreak plunged the world into a fatal pandemic condition [3]. One of the efforts to prevent the early spread of COVID-19 is an early forecast of new cases to effectively prepare facilities, map health workers and other resources, as well as optimize management strategies in handling patients. The prediction of disease spread plays an important role in control, treatment, and health decision-making [4]. Various prediction methods can be used to predict time series, including statistical, mathematical, as well as machine and deep learning approaches. According to Obeid (2023), aside from forecasting the spread of infectious diseases, prediction models can also be used for other purposes such as predicting products and ratios in electrical circuits [5]. These predictions offer valuable insight into the probable outcome of operating electric circuits. As noted by Algamal et al. (2023), prediction models are instrumental in assessing estimator performance and facilitating the simulation of new estimators slated for deployment in industries or manufacturing processes [6].

Several statistical-based prediction approaches have been used, including Linear Regression (LR), Multiple Linear Regression (MLR), Logistic Regression [7], Autoregressive Integrated Moving Average (ARIMA) [8], and Seasonal Autoregressive Integrated Moving Average (SARIMA) [[9], [10], [11], [12]]. However, due to non-linear components and non-stationary data flow characteristics, these methods have limitations. The iterative creation of predicted values can strain model performance [13]. The limitations include the ability to handle only non-linear correlations, necessitating complex data extraction processes, limited variables set, and challenges in achieving a high accuracy value [14]. Consequently, research has shifted to the use of the machine-learning approach to predict time series data. This approach enhances the ability to learn new information from data and develop capabilities to solve a problem, answer questions, and conclude data processing to provide accurate results [15]. Several models within this approach include Least Absolute Shrinkage and Selection Operator Regression (LASSO Regression), Exponential Smoothing (ES), Random Forest, and Support Vector Machine (SVM) [16]. These models can uncover hidden patterns and data relationships that may elude human observers. Machine learning models play a crucial role in predicting outcomes or making recommendations based on historical data [17]. Some models are complex and difficult to interpret, making it challenging to understand the rationale behind their forecasts or suggestions. These restrictions lead to the inability to accurately predict outcomes that differ from their training data [18]. One method to solve this problem is RNN, which can process sequential data and store information from the past, enabling the identification of trends [19]. A common RNN variant algorithm is LSTM, characterized by high performance in solving various tasks. LSTM provides neural networks with internal or short-term memory, making it particularly suitable for processing sequence-related problems such as speech classification, prediction, image subtitles, language translation, and handwriting recognition. It has been widely used to predict infectious diseases such as Dengue [[20], [21], [22]] and Malaria [23,24].

This research focused on predicting the number of COVID-19 cases based on time series data. The prediction works with time series data because the spread of the virus is associated with various interrelated variables that evolve. These variables include daily confirmed cases, fatalities, recoveries, and others. The cumulative confirmed cases represent the total number of cases in a specific time. This variable provides historical context and helps capture the scale of the outbreak as a whole. It plays a crucial role in understanding the course of the disease and serves as a basis for predicting the number of future cases. On the other hand, the confirmed cases on a given day represent the number of new infections detected. This variable aids in monitoring the progression and trends of disease spread, enabling the authorities to project the number of cases in the short term. The Lockdown Policy variable is used to determine the impact of interventions on disease dynamics. For example, the predictive results decreased cases when strict lockdown measure was implemented by the local government.

This paper proposes an LSTM-based COVID-19 confirmed case prediction model. The significant advantage is the ability to effectively predict long-term temporal dependencies. The primary inputs include confirmed cases, the growth rate of diagnoses, city closure status, longitude, latitude, and population of the region. These variables serve to capture data patterns by considering regional differences, thereby enabling the model to incorporate geographical contexts into its predictions. Meanwhile, the predicted outcome is the number of people infected.

This research proposes a more improved model with a superior fitting effect for large population areas. Moreover, its prediction effect is more accurate than the basic LSTM prediction algorithm and previous optimization models. The novelty of this research is to modify the output gate by maintaining the value of *o*_*t*_ < 0.5 and the value of *h*_*t*_ < 0.3. These adjustments increased the accuracy value of the model. The model was trained using data from four countries on different continents including China, Italy, Hong Kong, and Indonesia. These countries were selected due to their high daily cases and large population.

The organization of this paper is as follows: Section 2 explains the implementation of LSTM in previous research, specifically on the prediction of COVID-19 and optimization of accuracy. Section 3 describes the background of LSTM, the sequence of modification processes for improved model accuracy, and the testing process. Section 4 offers a comparison of prediction results using basic, previous, and proposed LSTM model, while Section 5 contains a summary of the research.

## 2 Related work

### 2.1 LSTM for infectious disease prediction

In the last three years, several investigations have been conducted on the implementation of LSTM for the prediction of infectious diseases, specifically COVID-19. Wahyuni et al. (2022) [25] carried out a comparison test of the accuracy of LSTM Ridge Regression, and Multiple Regression Linear models using the Indonesian COVID-19 dataset. The results showed that the accuracy was better than other previous models. Indriani et al. used the model to predict COVID-19 trends in Indonesia, the results showed a close relationship with real-time case data. Yang et al. (2020) [26] also predicted the disease trends in China and compared the accuracy with the Back Propagation (BP) model. The results showed that LSTM prediction results were more accurate than those of the BP model. Ko and Yoon also predicted the trend in South Korea over the next 4 weeks using LSTM and compared the results with those of the Susceptible–Infected–Recovered (SIR) model. LSTM prediction was found to be closer to real cases. Therefore, the LSTM model is highly recommended to predict short-term and long-term trends in the spread of COVID-19 due to its high accuracy in disease prediction [[27], [28], [29], [30], [31]].

### 2.2 Optimized LSTM prediction model for infectious disease prediction in recent years

Several optimized LSTM model for COVID-19 prediction has been conducted. Yan et al. (2020) proposed LSTM model by modifying MinMaxScaler process. The model was implemented to predict confirmed patients in several countries. The results showed an increase in accuracy by 2% from Basic LSTM model [32]. Improved MinMaxScaler was installed manually on the machine and only predicted data within a limited distance and small population. This indicates the need for revamping to extend predictions to considerable data distances and large populations. Additionally, Lee et al. (2020) successfully predicted an outbreak of Hepatitis A cases in Korea using LSTM [33], and the result exhibited high accuracy [34].

Choi and Lee (2018) adjusted the weighting stage and combined the weights dynamically to produce better predictive outcomes. During the last LSTM weighting, the value of W was dependent on the amount of data, namely 0 < γ ≤ 1,1 ≤ v ≤ k. In the new weighting model, a weight value of 0.3 was proposed. It reduces the complexity value in time series data prediction [35]. Arora et al. (2020) suggested structural improvements by adding LSTM cells to have more hidden layers. The model was used to predict the number of confirmed cases in 32 regions in India, resulting in a 3% difference from the actual data [36]. Wang et al. (2020) added automation to the model to predict confirmed cases in several countries. However, this automation must be reset for new datasets because COVID-19 data differs in every country [37]. This research proposed an optimized prediction model without resetting that can automatically be used for different data with large and small data distances.

## 3 Material and method

### 3.1 Data processing

Data on the number of confirmed cases from the previous day were used to predict trends for the next day. The data were collected from the official WHO website from day to day, and lockdown references implemented by the government at that time were considered. The decision to implement lockdown significantly affected the mobility of the population, thereby reducing the spread of confirmed cases. Regarding the lockdown data, longitude and latitude information were taken from Google and were presented in a clear format. The dataset included latitude, longitude, and population density data, which were used as predictive variables. Areas close to latitudes have higher population densities than distant areas. Therefore, population density is one of the variables that affect the increase in the number of COVID-19 cases. Data on the variable were obtained through the official website of the World Bank.

The data were processed using 3 scenarios. For scenario 1, the preprocessed data were entered into the Normalization window using MinMaxScaler, followed by prediction to obtain the number of confirmed cases. In the second scenario, the data were entered into the normalization window using a modified method, then the number of confirmed cases was predicted for the next 7 days.

In the third scenario, the data were entered into the normalization window and then predicted using an optimized model modified on the output gate. The output gate was adjusted to remain stable at <0.5 and control the information disseminated. This culminated in a higher accuracy value than the previous model. Function modifications on *o*_*t*_ were installed automatically, eliminating the need to manually disassemble the machine.

### 3.2 LSTM prediction model

**Fig. 1:**
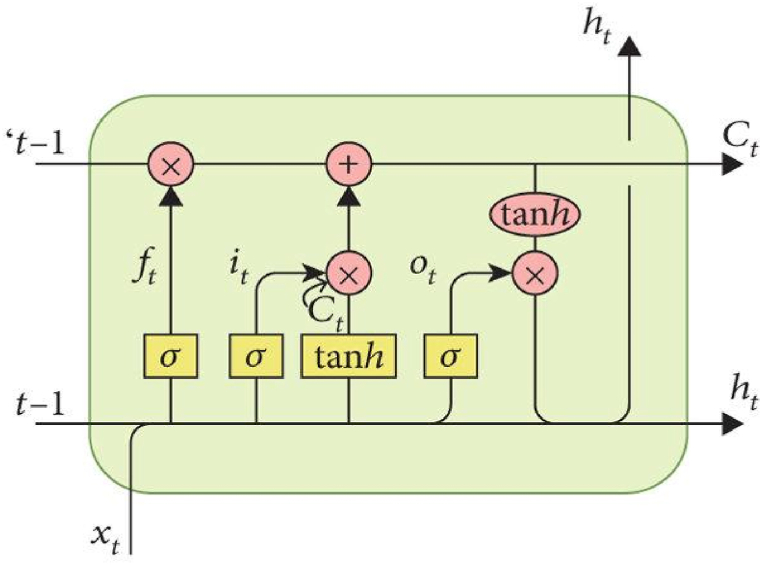
LSTM scheme.

LSTM has a higher accuracy in the prediction model than other RNN variants. In RNN, the iteration of the model only uses one simple single layer tanh. This layer in LSTM functions as a regulator of the flow of information on every input that enters the cell. Tanh also serves as a determinant of which information to retrieve, store, and forward for processing. It aims to make the input a number from −1 to 1. In the model, $x_{t-1}$ and $h_{t-1}$ the previous input and output were included along with the new one, while $h_{t+1}$ and $x_{t+1}$ served as the output and input after order *t* [38]. LSTM algorithm is presented in Fig. 1 and Equations (1)–(6).

(1) $$ft=\sigma (W_{f}.{[h}_{t-1},x_{t}]+b_{f}$$

(2) $$i_{t}=\sigma (W_{i}.\left[h_{t-1},x_{t}\right]+b_{i}$$

(3) $${\bar{C}}_{t}=\tanh\;(W_{c}.\left[h_{t-1},x_{t}\right]+b_{c}$$

(4) $$C_{t}=f_{t}*c_{t-1}+i_{t}*{\bar{C}}_{t}$$

(5) $$o_{t}=\sigma \left(W_{o}.\left[h_{t-1},x_{t}\right]+b_{o}\right)$$

(6) $$h_{t}=o_{t}*\tanh\left(C_{t}\right)$$

In this equation, $f_{t}$ = forgotten gate, $i_{t}=$ input gate, $C_{t}$ = cell state, $o_{t}$ = output gate, ***σ*** = sigmoid function, $W_{f}$ = weight value for the forget gate, $h_{\left(t-1\right)}$ = output value before the *t* order, $x_{t}$ = input value of the *t* order, and $b_{f}$ = bias value of the forget gate.

### 3.3 The proposed optimized LSTM model

**Fig. 2:**
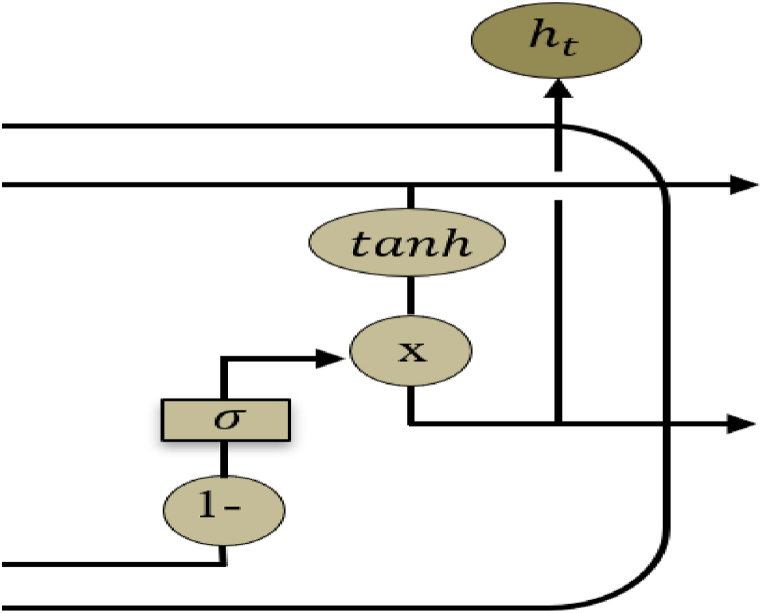
Output gate popLSTM Scheme.

The proposed optimized LSTM (popLSTM) model is presented in Fig. 2. The output layer on LSTM was optimized to obtain an output value of <0.5. The optimization step continued by putting the number 1 and subtracting by $o_{t}$. This will aid in predicting data for small and large populations with varying distances. In optimizing this model, spatial variables were added namely density, population, latitude, and longitude. The optimization is presented in Eqs. (7)–(12).

(7) $$1-ot=1-\;\sigma \left(W_{o}.\left[h_{t-1},x_{t}\right]+b_{o}\right)$$

where:

(8) $$\sigma \left(x\right)=\frac{1}{1+\epsilon^{-x}}$$

Accordingly,

(9) $$1-ot=\frac{\epsilon^{-W0\left[h_{t-1,}x_{t}\right]\;.\;}\epsilon^{-bo}}{1+\epsilon^{-W0\left[h_{t-1,}x_{t}\right]}.\epsilon^{-bo}}$$

The result shows that $o_{t}$ is

(10) $$ot=1-\frac{\epsilon^{-W0\left[h_{t-1,}x_{t}\right]\;.\;}\epsilon^{-bo}}{1+\epsilon^{-W0\left[h_{t-1,}x_{t}\right]}.\epsilon^{-bo}}$$

(11) $$ot=1-\frac{\epsilon^{-W0\left[h_{t-1,}x_{t}\right]\;.\;}\epsilon^{-bo}-\epsilon^{-W0\left[h_{t-1,}x_{t}\right]\;.\;}\epsilon^{-bo}}{1+\epsilon^{-W0\left[h_{t-1,}x_{t}\right]}.\epsilon^{-bo}}$$

(12) $$ot=\frac{1}{1+\epsilon^{-W0\left[h_{t-1,}x_{t}\right]}.\epsilon^{-bo}}$$

The final predicted value of LSTM showed dependency on the final value of $h_{t}$. Meanwhile, the last $h_{t}$ value depends on the previous $o_{t}$. In the context of this research, when the value of $o_{t}<$ 0.5, $h_{t}$ experienced a decrement by subtracting the value of 1 from $o_{t}$ or $1-\sigma \left(W_{o}.\left[h_{t-1},x_{t}\right]+b_{o}\right)$. Therefore, the last $o_{t}$ value decreased in number and the $h_{t}$ value automatically reduce also due to the multiplication of $o_{t}$ with tanh on $C_{t}$. The improved specific algorithm is as follows:

**Table:** 

Algorithm
**Input:** Data x is data in *t* period **Output**: data after processing
1 $o_{t}=\sigma \left(W_{o}.\left[h_{t-1},x_{t}\right]+b_{o}\right)$. 2 $\sigma \left(x\right)=\frac{1}{1+\epsilon^{-x}}$. 3 $h_{t}=o_{t}*\tanh\;\left(C_{t}\right)$. 4 **If**$o_{t}$ <0.5 **then** 5 $o_{t}=\frac{1}{1+\epsilon^{-W0\left[h_{t-1,}x_{t}\right]}.\epsilon^{-bo}}$. 6 end if

### 3.4 Proposed framework

**Fig. 3:**
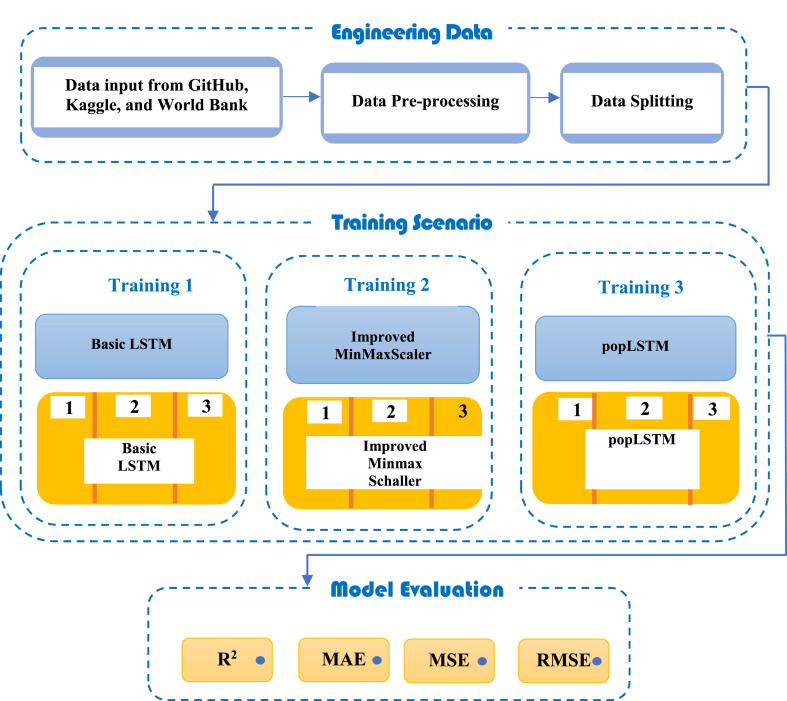
Architecture of the proposed methodology.

Fig. 3 shows the proposed framework for data training and testing on three different algorithms. There are 3 modules, namely:

Module I represents the process of data collection and preprocessing carried out with the following steps:

1. Step 1: Data were collected from four countries on different continents including China, Italy, Hong Kong, and Indonesia through several sources namely Github, Kaggle and the World Bank.
2. Step 2: Preprocessing and sharing of training as well as testing data with a proportion of 80% and 20% respectively.

Module II refers to the data training process conducted using 3 different models, namely Basic LSTM, Improved MinMaxScaler, and popLSTM.

Fig. 2 shows that the value of 1− was subtracted from the $o_{t}$ process. The process schema added one function to the output layer, automatically calculated by the model. The value of *o*_*t*_ becomes the input for *h*_*t.*_ Several steps taken in the optimization process included: The coronavirus exhibits an extended period of incubation, necessitating the consideration of many characteristics. Relying solely on LSTM for trend analysis is impractical, as the results are not ideal. The incubation period of the new coronavirus is more than 14 days, hence, basic data training was extended to 21 days to better capture future trends. The implementation steps of the experiment are as follows: Setting the model training, normalizing the data using MinMaxScaler, placing the number 1, and reducing it with tanh to get an *o*_*t*_ value below 0.5 automatically affecting the ht value. This position keeps the *h*_*t*_ value always less than 0.3.

### 3.5 Evaluation metrics

The compatibility scale calculates the discrepancy between the actual and expected values. R^2^ is often used to measure the goodness of fit for a model. Research indicates that a higher R^2^ value suggests a better model fit. Equations (13) until 18 were used to calculate R^2^, MAE, MSE, and RMSE. MAE is a common metric used to measure the average absolute difference between predicted and actual values in a data set. Meanwhile, MSE is used to calculate the average error in predictions. A smaller average squared error value indicates better model quality. RMSE functions to calculate the average root of the squared difference between the predicted and the actual value [39].

(13) $$R^{2}=\frac{SSregression}{SStotal}$$

where SSregression is:

(14) $$\sum_{i=1}^{n}{\left({\hat{y}}_{i}-\bar{y}\right)}^{2}$$

where $\hat{y_{i}}$ is the predicted value at the i-th point, $\bar{y}$ is the average of the actual values.

(15) $$\sum_{i=1}^{n}{\left(y_{i}-\bar{y}\right)}^{2}$$

where $y_{i}$ is the actual value to-*I* point and $\bar{y}$ is the average of the actual values.

(16) $$MAE=\frac{1}{n}\sum_{j=1}^{n}\left|y_{j}-\hat{y_{j}}\right|$$

where n is the amount of data, $y_{j}$ is the actual value at the point to-*j*, and $\hat{y}$ is prediction value.

(17) $$MSE=\frac{1}{n}\sum_{i=1}^{n}{\left(y_{i}-\hat{y_{i}}\right)}^{2}$$

where $y_{i}$ is the actual value at the point to*-i* and $\hat{y_{i}}$ is the predicted value of the point to *i*.

(18) $$RMSE=\sqrt{\frac{1}{n}}\sum_{i=1}^{n}{(y_{i}-\hat{y_{i})}}^{2}$$

Module III is the process of testing models using R^2^, MAE, MSE, and RMSE.

### 3.6 Parameters

• Step I: Time series length Setting. The duration of data training was set for 21 days, referring to the incubation period of the COVID-19 virus.
• Step II: The forecast for the new confirmed case was set for 7 days.
• Step III: The output model was set in time series.

## 4 Result and discussion

This section discusses the steps taken and the results obtained from popLSTM. It compares the accuracy of Basic LSTM, improved MinmaxScaler, and popLSTM model. This research used data from four countries across various continents, including Hong Kong, Italy, South Korea, and Indonesia. These countries were selected due to their significant upward trend in daily COVID-19 cases as of January 20, 2020. Furthermore, the dataset consisted of four time series sets, including cumulative diagnosed, confirmed, and additional daily cases, as well as lockdown policies. Each dataset served as the foundation for optimizing LSTM.

### 4.1 Dataset

The dataset used was derived from Ref. [32] and available at https://github.com/CSSEGISandData/COVID-19, while the latitude and longitude data finder is available at https://www.latlong.net/. Furthermore, the data training was conducted for 21 days, and predictions were made for the next 7 days, from April 10 to 16, 2020. To assess the accuracy and reliability of the model, multiple countries were selected for data collection. The variables used as predictive parameters included the cumulative diagnosed, confirmed, and additional daily cases, as well as lockdown policies. The Lockdown data are available at https://www.kaggle.com/datasets/jcyzag/covid19-lockdown-dates-by-country. Each dataset row included latitude, longitude, and population density data for the respective countries. The population density data are available at https://data.worldbank.org/indicator/EN.POP.DNST?most_recent_value_desc=true. The trend of new cases was predicted in the following days with an optimized model. Prediction was carried out independently by inputting the relevant time series data in the modified model. Using Google Collaboratory tools and several libraries from the scikit-learn and Tensorflow to show the corresponding outcomes between the real and predicted data, a trained model predicted cases for the next 7 days, from April 10 to 16, 2020. The experiments were intended to evaluate how well popLSTM predicted COVID-19 cases in Italy, Hong Kong, South Korea, and Indonesia.

### 4.2 Experiment result

**Fig. 4:**
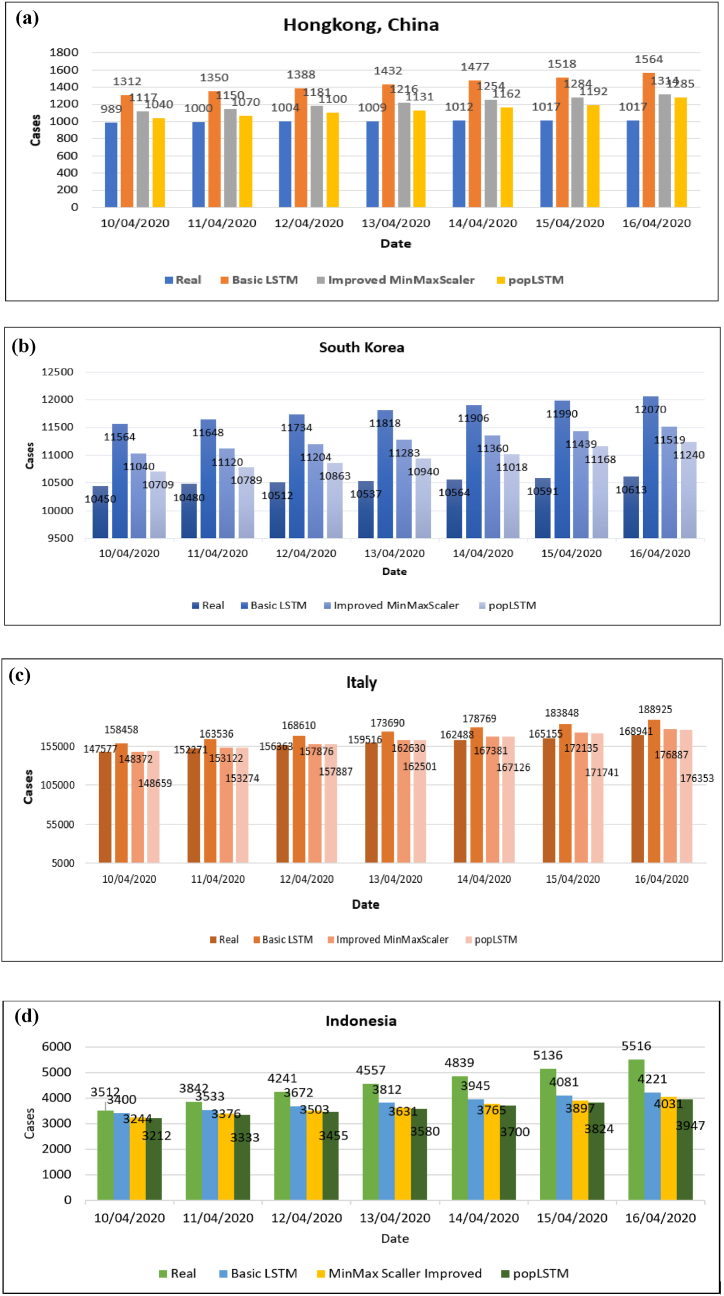
(a)–(d) Comparison between the number of confirmed cases and predicted cases by different models.

The predicted outcomes of the four countries using different models are presented in Table 1 and Fig. 4(a–d).

**Table 1:** Comparison of Chi-square test results.

Model	Evaluation	Mean Rank	*p-value*
popLSTM	MSE	115.92	0.0017
	RMSE	111.85	0.0018
	MAE	116.12	0.0028
Improved MinMaxScaler	MSE	119.65	0.0019
	RMSE	121.61	0.0037
	MAE	118.06	0.0050
Basic LSTM	MSE	143.93	0.008
	RMSE	146.05	0.027
	MAE	145.32	0.039

### 4.3 Discussion

This section explains the prediction results from four countries in Europe and Asia namely Italy, Hong Kong, South Korea, and Indonesia. The policy differences between the two continents were considered for testing the strength of the developed model. The training in this research consisted of three scenarios. In the first scenario, Basic LSTM was trained using normalization MinMaxScaler, while in the second scenario, improved MinMaxScaler was used. For the last scenario, popLSTM was implemented. Subsequently, the outcomes of popLSTM trial were used to predict the number of confirmed patients.

Based on Fig. 4, popLSTM had a predictive value closer to the actual one. Fig. 4(a) shows the prediction result for Hong Kong, China, with popLSTM having an average difference of 8% from the real value. The average difference of Basic LSTM, MinMaxScaler, and popLSTM was 35%, 14%, and 8%, respectively, in the process of predicting the number of confirmed cases in Hong Kong, China. These differences were attributed to many factors, including the random method used to collect training data, resulting in variations in prediction results. This is one of the weaknesses of deep learning models in making predictions, specifically in the data training process.

Fig. 4(b) shows the prediction result for South Korea. On average, popLSTM performed better than the other two in forecasting the number of confirmed cases. This was indicated by the lower average difference (2%) between the predicted and actual values compared to improved MinMaxScaler (5%) and Basic LSTM (10%).

**Fig. 5:**
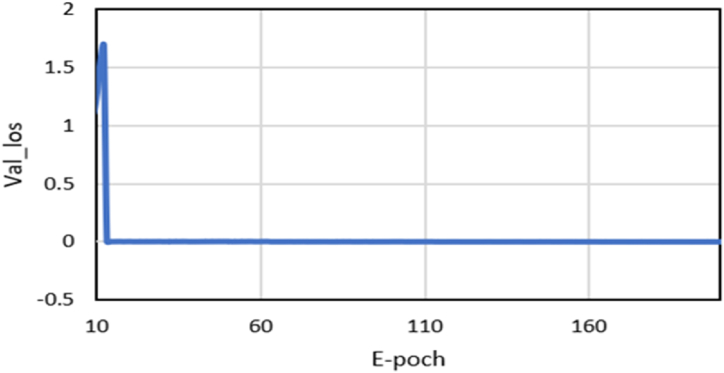
Validation loss of popLSTM versus the number of epochs.

Fig. 4(c) presents the predicted result for Italy. Basic LSTM had an average difference of 10% in its forecast results, while MinMaxScaler and popLSTM was 3% and 1%, respectively. This implies that popLSTM is suitable for predicting the number of cases in Italy. Fig. 4(d) represents the mean discrepancy in predicted results for Indonesia. Basic LSTM had a difference of 13.3%, while MinMaxScaler and popLSTM had values of 12.6% and 11.8%, respectively. Table 2 shows the predicted results of all training scenarios. popLSTM was found to be more effective in forecasting the number of cases across different countries. During the training process, popLSTM showed a decrease in the Val_loss value, which remained consistent despite the large epoch value, showing excellent performance. The epoch process is depicted in Fig. 5.

**Table 2:** Comparison between real cases and predicted results.

Countries	Date	Real Cases	Basic LSTM	Improved MinMaxScaler	popLSTM
Hongkong, China	10/04/2020	989	1312	1117	1040
	11/04/2020	1000	1350	1150	1070
	12/04/2020	1004	1388	1181	1100
	13/04/2020	1009	1432	1216	1131
	14/04/2020	1012	1477	1254	1162
	15/04/2020	1017	1518	1284	1192
	16/04/2020	1017	1564	1314	1285
South Korea	April 10, 10/04/2020	10,450	11,564	11,040	10,709
	11/04/2020	10,480	11,648	11,120	10,789
	12/04/2020	10,512	11,734	11,204	10,863
	13/04/2020	10,537	11,818	11,283	10,940
	14/04/2020	10,564	11,906	11,360	11,018
	15/04/2020	10,591	11,990	11,439	11,168
	16/04/2020	10,613	12,070	11,519	11,240
Italy	10/04/2020	147,577	158,458	148,372	148,659
	11/04/2020	152,271	163,536	153,122	153,274
	12/04/2020	156,363	168,610	157,876	157,887
	13/04/2020	159,516	173,690	162,630	162,501
	14/04/2020	162,488	178,769	167,381	167,126
	15/04/2020	165,155	183,848	172,135	171,741
	16/04/2020	168,941	188,925	176,887	176,353
Indonesia	10/04/2020	3512	3400	3244	3212
	11/04/2020	3842	3533	3376	3333
	12/04/2020	4241	3672	3503	3455
	13/04/2020	4557	3812	3631	3580
	14/04/2020	4839	3945	3765	3700
	15/04/2020	5136	4081	3897	3824
	16/04/2020	5516	4221	4031	3947

### 4.4 Statistical analysis

To determine the significance of popLSTM, statistical analysis was used to test the accuracy of all three models. A normality test was carried out on the training data from the three models, using parametric or non-parametric statistics. The test was performed using the *t-test* for parametric and the Chi-Square test for non-parametric. The Chi-Square test was used to determine the performance of popLSTM, MinMaxScaler, and Basic LSTM. The results obtained are presented in Table 1 below.

In this test, a confidence level of 95% and α = 5% was used, while Table 1 shows that the overall p-value was <0.05. Based on the results, H0 was rejected and Ha was accepted. This means that there was a significant improvement in popLSTM compared to the other models. In popLSTM, the p-value for MSE test was 0.0017 (<0.05), indicating a decrease in error and a significant increase in accuracy.

### 4.5 Evaluation result

The comparison between the prediction results of all models are shown in Table 2 and the model accuracy evaluation results obtained using R^2^, MAE, MSE, and RMSE metrics are presented in Table 3.

**Table 3:** Performance evaluation matrices of evaluated models.

Model	Dataset	R^2^	MAE	MSE	RMSE
Basic LSTM [25]	Hongkong	0.9914	0.398	12205.83	110.48
	South Korea	0.9898	0.399	382346.49	618.34
	Italy	0.9916	0.51	26324780.46	5130.77
	Indonesia	0.9939	0.4667	26324780.46	83.12
Improved MinMaxScaler [32]	Hongkong	0.992	0.3894	1410	37.6
	South Korea	0.9904	0.3629	395.46	19.89
	Italy	0.9922	0.3232	2371324.75	1539.91
	Indonesia	0.997	0.3334	1752.37	41.86
popLSTM	Hongkong	0.9951	0.3779	395,46	19.89
	South Korea	0.994	0.334	15618.17	124.97
	Italy	0.9953	0.3235	264382.59	514.18
	Indonesia	0.997	0.3137	302.75	17.4

We analyzed the prediction results of the three models, namely Basic LSTM, improved MinMaxScaler, and popLSTM that are presented in Table 2. The difference between the number of predicted cases using popLSTM for 7 days and the number of real cases were only 2%. So this model has a better performance compared to other models.

Table 3 presents the differences in model testing results. Based on the R2 in each country, popLSTM had a precision difference of 4% greater than the actual value. This was evidenced by the precision value within each country, with a distinction of 0.04 points. Based on RMSE, the model had a small value compared to Basic LSTM and improved MinMax Scaler. These prediction results are very important in tracking the emergence of new variants of the COVID-19 virus by the government and other related parties. Currently, a new variant of the virus has been reported in 10 countries including within Asia.

**Fig. 6:**
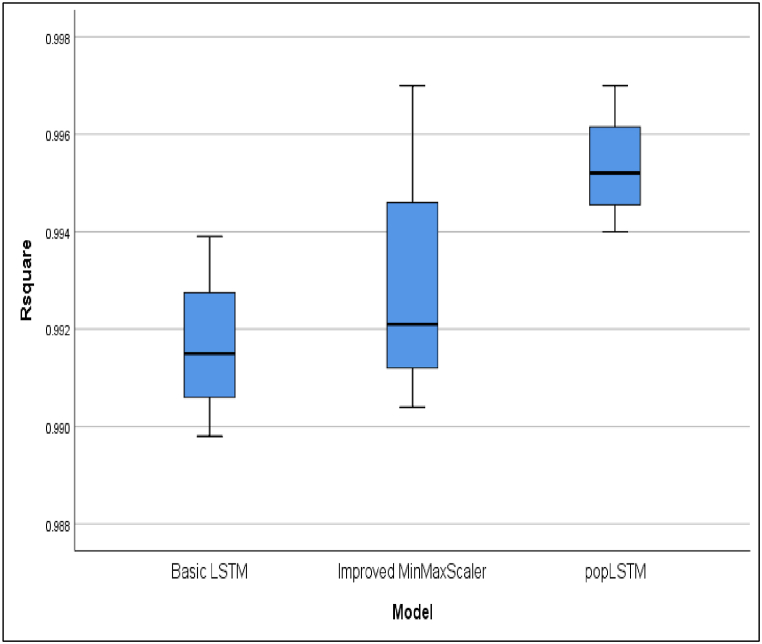
Comparison between the R square values of different models.

**Fig. 7:**
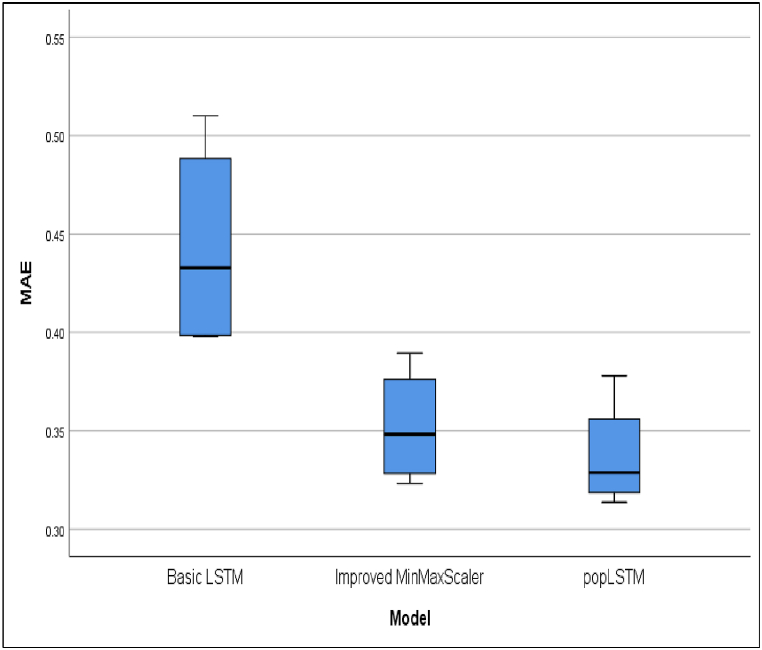
Comparison between the MAE of different models.

**Fig. 8:**
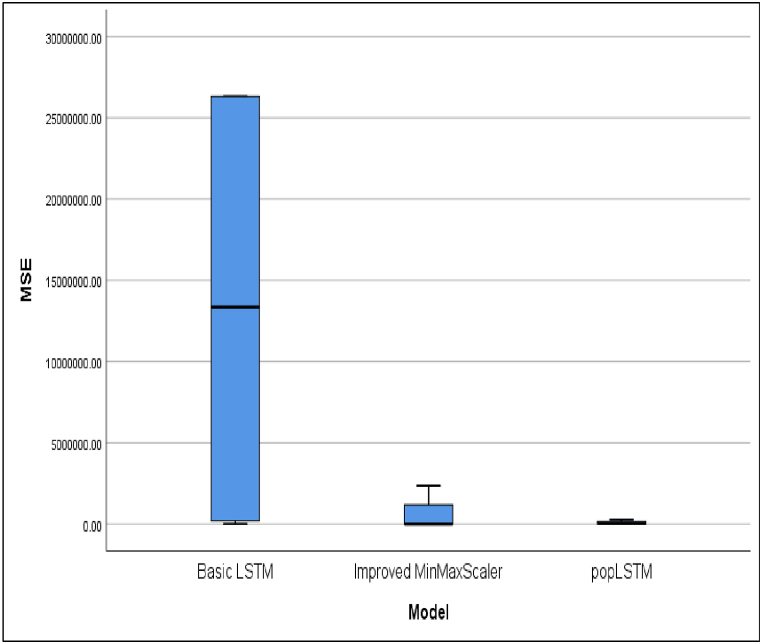
Comparison between the MSE of different models.

**Fig. 9:**
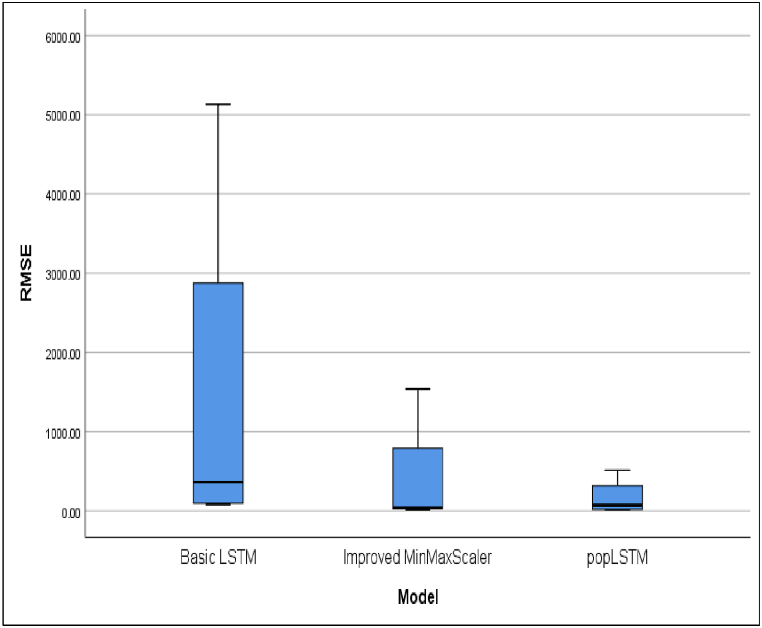
Comparison of RMSE values across models.

This development underscores the urgency of making predictions based on daily case data. These predictions are instrumental in facilitating early control efforts to reduce the risk of death and prepare healthcare facilities for the community. Table 3 presents a comparative analysis of R^2^ or R Square values for cases across four countries. The average of the performance matrices of evaluated models are presented in Table 4. The performance different models in terms of R^2^, MAE, MSE and RMSE are shown in Fig. 6, Fig. 7, Fig. 8, Fig. 9, respectively.

**Table 4:** The average of performance matrices of different models'.

Model	Rsquare	MAE	MSE	RMSE
Basic LSTM	0,9917	0,4434	13,261,028,3100	1485,6775
Improved MinMaxScaler	0,9929	0,3522	593,720,6450	409,8150
popLSTM	0,9954	0,3373	79,962,3775	169,1100

The differences in accuracy presented in Fig. 6 were attributed to the intervention of spatial variables in popLSTM. Therefore, popLSTM can be recommended as a prediction tool for COVID-19. As shown in Fig. 7, popLSTM had a lower MAE than the other models. The error value decreased with a lower MAE and the average was 0.44, 0.35, and 0.33 for Basic LSTM, improved MinMaxScaler, and popLSTM, respectively. Based on these results, popLSTM had a better performance than the other models regarding error levels.

Fig. 8 shows the comparison result of MSE value for all models tested. Compared to the values from other models, MSE for popLSTM across the four countries was very low.

The RMSE values of different models are depicted in Fig. 9.

Differences in RMSE values for popLSTM were smaller than in other models. This indicated that the predicted results were accurate. Based on RMSE test, popLSTM was recommended for predicting infectious diseases. The results showed that popLSTM with the inclusion of spatial variables performed better than the others, showing greater effectiveness in predicting the number of COVID-19 cases.

Certain limitations were associated with this research including limited datasets and differences in datasets. The number of datasets obtained during the pandemic greatly affected the experiment results.

## 5 Conclusion

In conclusion, this research proposed the use of popLSTM in the deep learning approach to overcome the inaccuracies associated with predicting the number of confirmed COVID-19 cases using Basic LSTM. The model produced better predictive results using training data from four different countries. To enhance the model accuracy, a method was used where the output gate was set to <0.5, by subtracting the value of 1. The data training time was 21 days in line with the extended incubation period of the virus. This consisted of 14-day and 7-day data training as a short-term prediction period.

The prediction variables were gathered from several publicly available data sources, namely confirmed cases, the growth rate of diagnoses, city closure status, longitude, latitude, and population density. Latitude, longitude, and population density were included to enhance accuracy and capture the trend of COVID-19 spread. These variables served as inputs to the prediction models, while the number of people infected was the outcome. The accuracy was calculated based on the average training data for the three models, namely Basic LSTM, improved MinMaxScaler, and popLSTM. The experimental results showed that popLSTM had better accuracy than the others. The limitations of this research included differences in datasets and data spacing. These variations can lead to adjustments in the model during the prediction process, necessitating a reset. The model is best suited for large datasets with substantial data distances. Future research should explore other time series prediction algorithms capable of automatic machine adjustment to reduce training time.

## Funding

This work was supported by Satya Wacana Christian University Indonesia.

## Data availability statement

Data availability in https://data.mendeley.com/v1/datasets/publish-confirmation/4j22vtxxf2/3.

## CRediT authorship contribution statement

**Irwan Sembiring:** Validation. **Sri Ngudi Wahyuni:** Writing – review & editing, Writing – original draft, Visualization, Methodology, Data curation, Conceptualization. **Eko Sediyono:** Methodology, Funding acquisition, Formal analysis.

## Declaration of competing interest

The authors declare that they have no known competing financial interests or personal relationships that could have appeared to influence the work reported in this paper.
